# One-stage vs. two-stage thoracoscopic surgery for synchronous bilateral pulmonary nodules: a systematic review and meta-analysis

**DOI:** 10.3389/fsurg.2025.1755084

**Published:** 2026-01-21

**Authors:** Rachid Eduardo Noleto da Nobrega Oliveira, Guilherme Franceschini Machado, Isabella Cabianca Moriguchi Caetano Salvador, Paula Duarte D´Ambrosio, Lucas Monteiro Delgado, Felipe S. Passos, Tulio Caldonazo

**Affiliations:** 1 Department of Thoracic Surgery, Barretos Cancer Center, Barretos, Brazil; 2 Hospital Santa Marcelina, São Paulo, Brazil; 3 University Anhembi Morumbi, São Paulo, Brazil; 4 Department Thoracic Surgery, Hospital das Clínicas, Faculty of Medicine, University of São Paulo (HCFMUSP), São Paulo, Brazil; 5 Universidade Federal de Minas Gerais, Belo Horizonte, Brazil; 6 Department of Thoracic Surgery, Hospital MaterDei, Salvador, Brazil; 7 Department of Cardiothoracic Surgery, Jena University Hospital, Jena, Germany

**Keywords:** bilateral lung resection, cancer, one-stage surgery, pulmonary nodules, thoracoscopic surgery

## Abstract

**Introduction:**

The optimal surgical strategy for synchronous bilateral pulmonary nodules remains unclear. One-stage bilateral resections may offer logistical and clinical advantages, but safety concerns persist regarding bilateral complications.

**Methods:**

We conducted a systematic review and meta-analysis of studies comparing one-stage vs. two-stage pulmonary resections in adult patients with synchronous bilateral nodules. Ten observational studies were included, encompassing 1,015 patients. Continuous outcomes were assessed using mean differences (MDs) and binary outcomes with odds ratios (ORs), applying DerSimonian and Laird random-effects models. Subgroup and meta-regression analyses were performed. Statistical analyses were conducted using R software (v4.4.1).

**Results:**

One-stage resection was associated with significantly reduced operative time (MD −24.36 min; 95% CI −40.59 to −8.13), shorter hospital stay (MD −2.79 days; 95% CI −4.25 to −1.33), and lower direct surgical costs (MD −5,543.73 USD; 95% CI −6,601.05 to −4,486.40). No significant differences were observed in intraoperative blood loss, persistent air leak, or arrhythmia. Subgroup analysis revealed that the type of pulmonary lesion influenced hospital stay, while meta-regression showed no effect of lobectomy rate.

**Conclusions:**

One-stage bilateral resection demonstrates greater efficiency without increased morbidity, supporting its use in experienced centers. These findings suggest that a single-anesthetic approach may facilitate earlier recovery and timely systemic therapy in selected patients.

**Systematic Review Registration:**

https://www.crd.york.ac.uk/PROSPERO/view/CRD420251048804, identifier: CRD420251048804.

## 1 Introduction

Advances in imaging technologies and lung cancer screening have led to a substantial increase in the detection of bilateral pulmonary nodules, many of which are clinically indeterminate or carry a high suspicion of malignancy. These include both synchronous multiple primary lung cancers (SMPLCs) and bilateral metastatic or presumed malignant lesions (1–3). When surgical resection is considered appropriate, thoracic surgeons must choose between a one-stage or two-stage approach, balancing oncologic efficacy with operative safety.

One-stage bilateral pulmonary resection, particularly when performed using minimally invasive techniques such as video-assisted thoracoscopic surgery (VATS), may offer several advantages. These include a single exposure to anesthesia, shorter cumulative hospital stay, reduced overall cost, and earlier initiation of adjuvant therapies where indicated (4–6). However, concerns persist regarding potential increases in operative time, technical complexity, and the risk of bilateral complications such as prolonged air leak or respiratory compromise (7–9).

While most early studies have focused on SMPLCs, more recent evidence suggests that one-stage resections may also be feasible and beneficial in selected patients with bilateral indeterminate or metastatic nodules (10–12). For instance, Yao et al. demonstrated the safety and effectiveness of one-stage bilateral VATS in patients with multiple small nodules, reporting similar complication rates and shorter overall hospitalization when compared to two-stage surgery (13). Yang et al., Wang et al., and Han et al. provided robust comparative data from high-volume centers supporting the oncologic and perioperative safety of one-stage resection strategies (14–16).

Given the expanding indications for surgical intervention in bilateral pulmonary nodules and the absence of consensus regarding the optimal surgical approach, we conducted a systematic review and meta-analysis comparing one-stage and two-stage resections. Our objective was to synthesize available evidence across diverse populations, including SMPLCs, bilateral lung metastases, and provide data-driven insights to guide surgical decision-making.

## 2 Methods

This systematic review and meta-analysis followed the Preferred Reporting Items for Systematic Reviews and Meta-Analysis (PRISMA) guidelines (17). The study protocol was registered in the International Prospective Register of Systematic Reviews (PROSPERO) under the registration number CRD420251048804.

### 2.1 Search strategy and data extraction

We systematically searched PubMed, Embase, and Cochrane Library from inception to May 01, 2025, the search strategy is detailed in Supplementary Material. Studies were initially imported into EndNote for deduplication and then into Zotero for screening. Each study was reviewed in Zotero at the title, abstract, and full-text levels based on inclusion and exclusion criteria. Reference sections of the included studies were evaluated to include additional studies. Two authors (F.B. and I.C.M.C.S.) independently extracted baseline characteristics and outcome data. Disagreements were resolved by consensus with a third author (R.E.N.N.O.).

### 2.2 Eligibility criteria

Studies with the following criteria were included: (1) retrospective studies or randomized controlled trials (RCTs); (2) comparing one-stage vs. two-stage thoracoscopic surgery; (3) enrolling adult patients (>18 years old) with synchronous bilateral pulmonary nodules; and (4) reporting at least one outcome of interest. We excluded articles with overlapping populations or treatments. No language filters were applied.

### 2.3 Endpoints

The endpoints of interest were: (1) operative time, (2) hospital length of stay (LOS), (3) surgical costs, (4) blood loss, (5) arrhythmia, and (6) persistent air leak.

### 2.4 Risk of bias and publication bias

The risk of bias of non-randomized studies was assessed by Risk of Bias In Non-randomised Studies—of Interventions (ROBINS-I) (18). Each study received a low, moderate, serious, or critical risk of bias in seven domains: confounding; selection of participants; classification of interventions; deviations from intended interventions; missing data; measurement of outcomes; and selection of reported results. Two independent authors conducted the risk of bias assessment (F.B. and I.C.M.C.S.), and disagreements were resolved unanimously with the senior author (R.E.N.N.O.). In addition to Egger's test, funnel plots and meta regression were used to assess publication bias.

### 2.5 Statistical analysis

The treatment effects for continuous outcomes were compared using mean differences (MDs) and binary endpoints were evaluated using odds ratios (ORs), with 95% confidence intervals (CIs). Heterogeneity was assessed with the Cochran *Q*-test and *I*^2^ statistics; *p* values <0.10 and *I*^2^ values >25% were considered to indicate significance for between-study heterogeneity (19). DerSimonian and Laird random effects models were used for all endpoints (20). In addition, we performed a meta-regression for hospital LOS to determine whether its effect size interacted with the proportion of patients who underwent lobectomy in each study (40, 41). The Cochrane Handbook for Systematic Reviews of Interventions was used for data handling and conversion (19). Statistical analyses were performed using R software, version 4.4.1 (R Foundation for Statistical Computing, Vienna, Austria).

## 3 Results

### 3.1 Study characteristics

**Figure 1:**
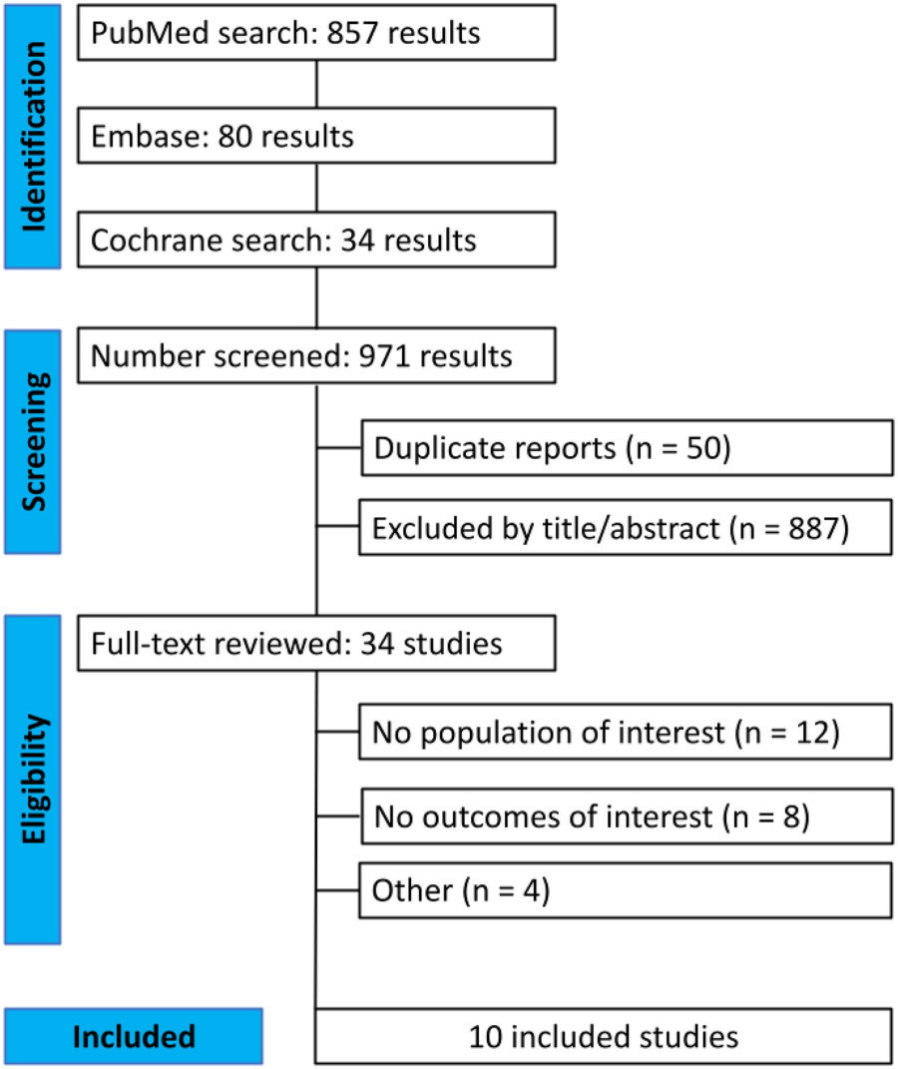
Preferred reporting items for systematic review and meta-analysis (PRISMA) flow diagram of study screening and selection.

Figure 1 shows the PRISMA flow diagram outlining the study selection process. A total of 971 studies were retrieved from the systematic search, of which 10 met the criteria for inclusion in the final analysis (13–16, 21–23, 42–44). Included studies were published between 2013 and 2025. All studies used registry data and originated from China, Hong Kong, Japan and South Korea.

### 3.2 Patient characteristics

Table 1 shows the individual study information. Ten observational studies were included in this meta-analysis, of which 9 were retrospective and one was prospective, encompassing 1,015 patients. From this group, 552 patients underwent single-stage lung resection and 463 a two-stage resection. The number of patients in each study ranged from 59 to 237. The age ranged from 52.8 to 67 years, with the percentage of male patients varying from 24.7% to 55.5%.

**Table 1:** Summary of baseline characteristics of included studies.

Study	Country	Time frame	Type of study	Sample size, SS/TS, *n*	Age, SS/TS, median or mean	Male, SS/TS, *n* (%)	Lobectomy (%)
Chen, 2024 (42)	Hong Kong	2021–2023	Retrospective	27/56	65.8/64.9^b^	8 (29.7)/27 (48.2)	45.00
Han, 2014 (43)	South Korea	2006–2013	Retrospective	52/9	54^a^^,c^	35^c^	0.00
Han, 2025 (16)	China	2019–2022	Retrospective	48/48	56.9/57.8^b^	15 (31.3)/14 (29.2)	44.79
Lan, 2020 (21)	China	2010–2018	Retrospective	54/42	56.2/58.9^b^	28 (52)/25 (59)	74.00
Ohtaka, 2013 (22)	Japan	2001–2007	Retrospective	67/11	66/67^a^	30 (44.8)/5 (45.5)	100.00
Wang, 2023 (15)	China	2014–2020	Retrospective	36/23	56.4/54.8^b^	20 (55.5)/10 (43.5)	54.24
Xu, 2022 (44)	China	2021	Prospective non-randomized	40/40	52.8/56.9^b^	12 (30)/17 (42.5)	75.00
Yang, 2024 (14)	China	2017–2023	Retrospective	158/79	58/58^a^	39 (24.7)/21 (26.6)	59.49
Yao, 2016 (13)	China	2009–2014	Retrospective	29/89	56.9/60^b^	14 (48.3)/49 (55.1)	84.75
Zheng, 2021 (23)	China	2013–2017	Retrospective	41/66	55.9/59.3^b^	16 (39)/35 (53)	100.00

SS, single stage; TS, two stage.

a Median.

b Mean.

c The data presented were for the entire population.

### 3.3 Pooled analysis of all studies

#### 3.3.1 Operative outcomes

**Figure 2:**
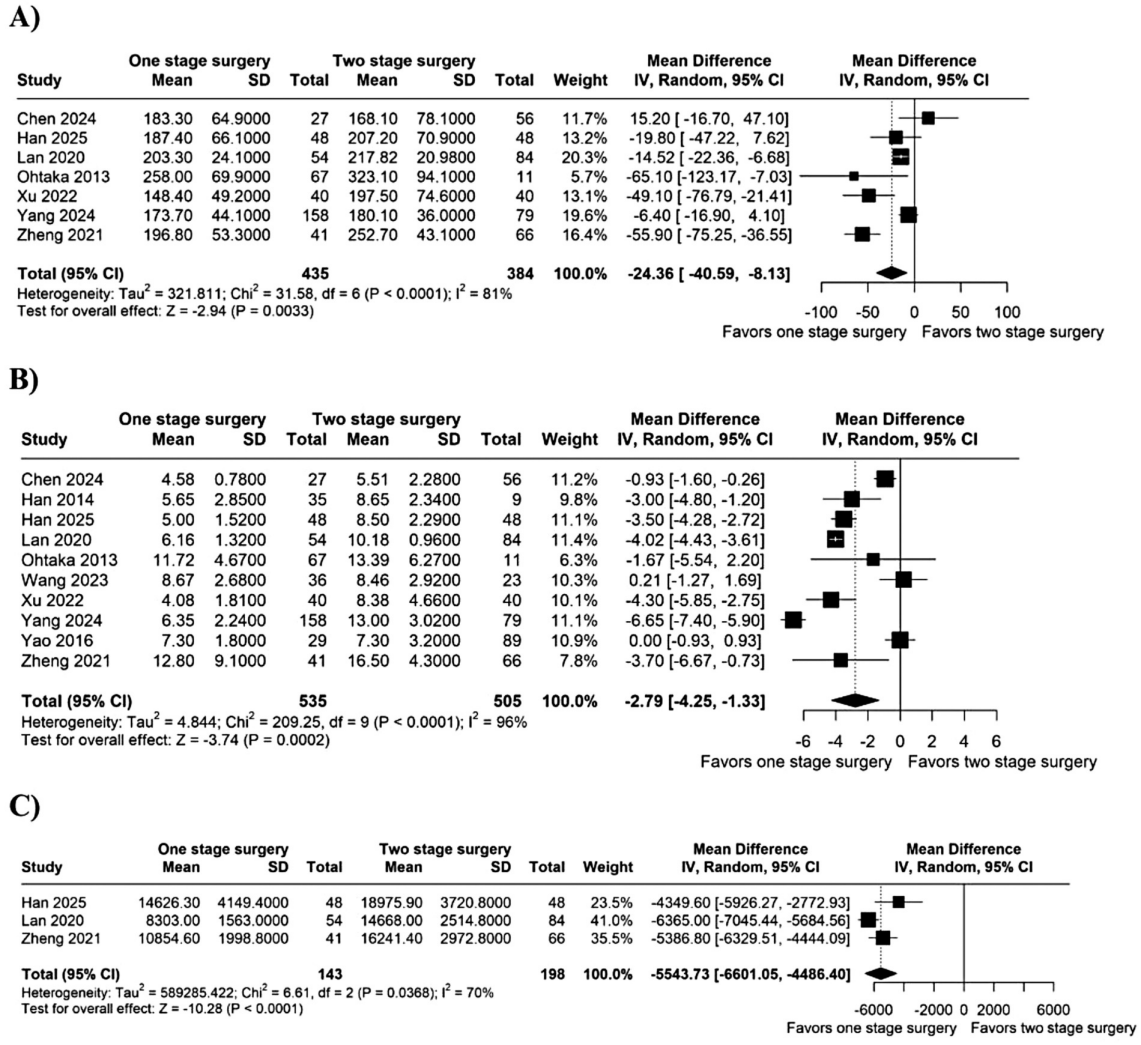
Forest plot of **(A)** operative time; **(B)** hospital LOS; and **(C)** cost. CI, confidence interval; IV, inverse variance; SD, standard deviation.

Operative time was significantly shorter in the single-stage group (MD −24.36 min; 95% CI −40.59 to −8.13; *I*^2^ = 81%; *p* < 0.05; Figure 2A). Hospital LOS was also reduced in the single-stage group (MD −2.79 days; 95% CI −4.25 to −1.33; *I*^2^ = 96%; *p* < 0.05; Figure 2B). Surgical costs were lower with the single-stage approach (MD −5,543.73 USD; 95% CI −6,601.05 to −4,486.40; *I*^2^ = 70%; *p* < 0.05; Figure 2C).

**Figure 3:**
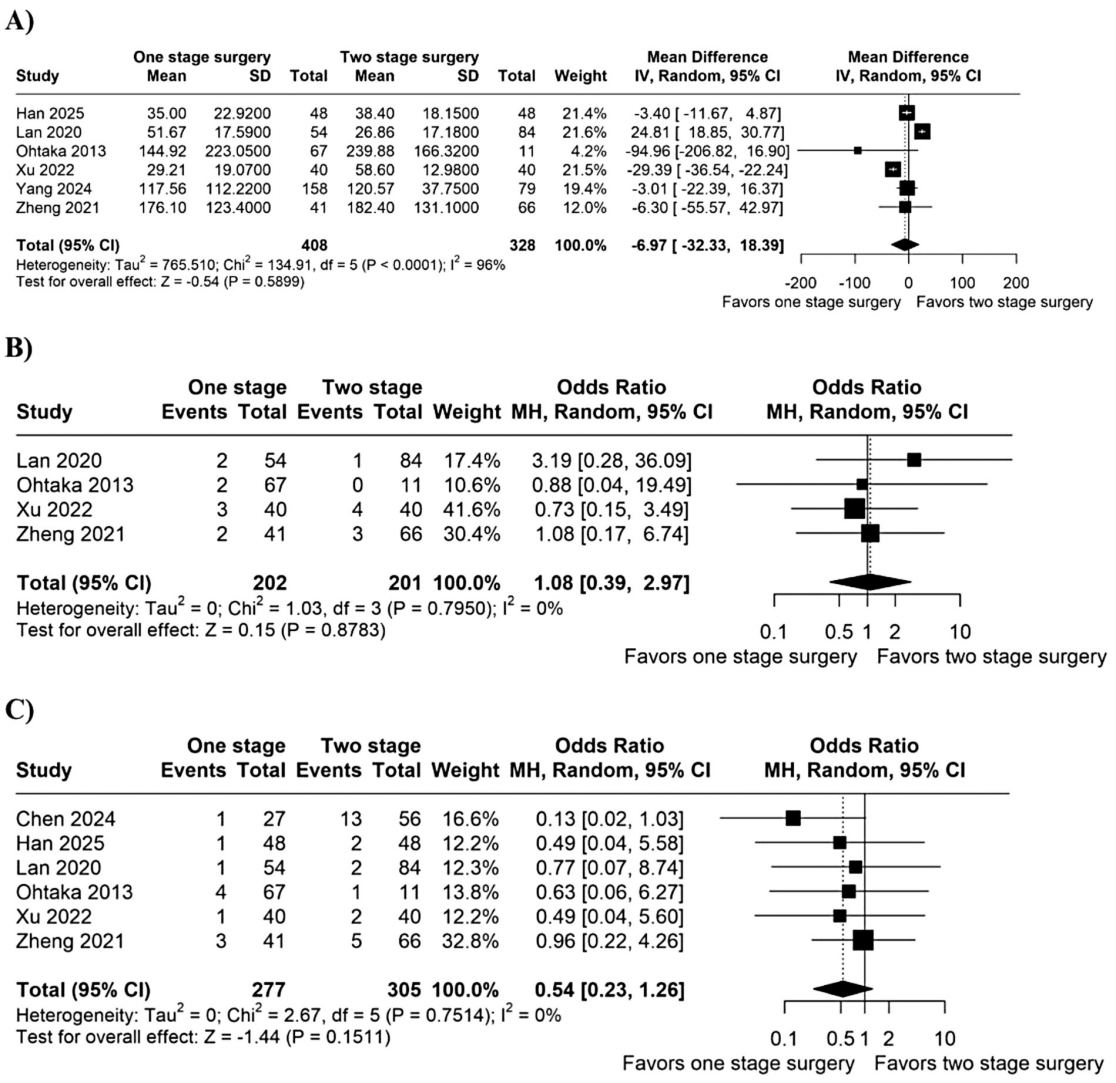
Forest plot of **(A)** blood loss; **(B)** arrhythmia; and **(C)** persistent air leak. CI, confidence interval; IV, inverse variance; MH, Mantel-Haenszel; SD, standard deviation.

There were no significant differences between groups for blood loss (MD −6.97 mL; 95% CI −32.33 to 18.39; *I*^2^ = 96%; *p* = 0.59; Figure 3A), arrhythmia (OR 1.08; 95% CI 0.39–2.97; *I*^2^ = 0%; *p* = 0.88; Figure 3B), or persistent air leak (OR 0.54; 95% CI 0.23–1.26; *I*^2^ = 0%; *p* = 0.15; Figure 3C).

#### 3.3.2 Subgroup analysis

Subgroup analyses disclose a significant difference in effect size attributable to lung lesion etiology (combined lung lesion, lung metastasis, or primary lung cancer) in hospital LOS (chi^2^ = 30.22; df = 2; *p* < 0.0001; Supplementary Figure S1).

### 3.4 Sensitivity analyses

We performed a leave-one-out sensitivity analysis for hospital LOS, revealing the stability of the result found, with no changes in the significance of the outcome. The leave-one-out sensitivity analysis plot is detailed in Supplementary Figure S2.

### 3.5 Quality assessment and publication bias

**Figure 4:**
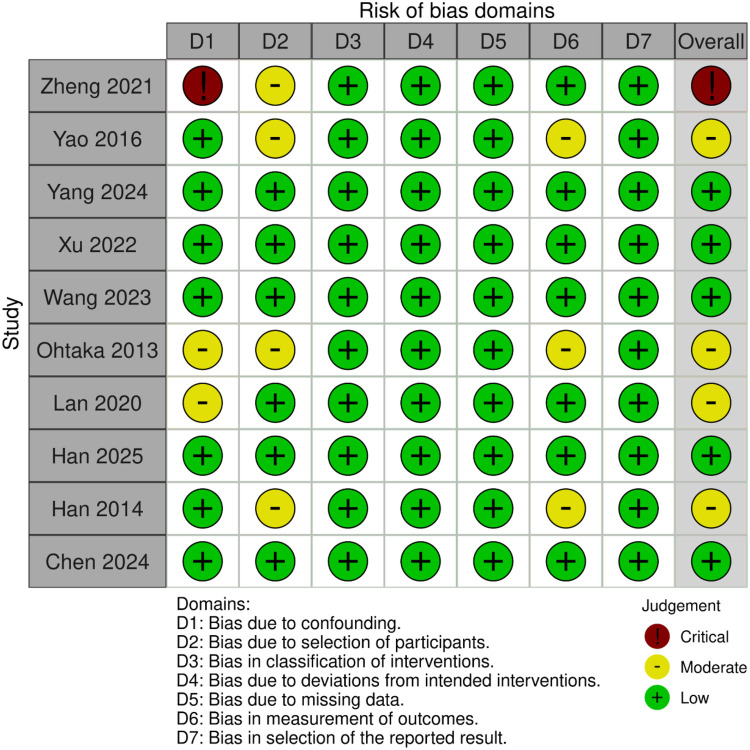
Critical appraisal of studies according to risk of bias in non-randomized studies of interventions (ROBINS-I).

Risk-of-bias was appraised with ROBINS-I across seven domains, Figure 4. Bias due to confounding was judged low in 7 of 10 studies, moderate in 2, and critical in 1 (21–23). Bias from participant selection was low in 6 studies and moderate in the remaining 4. All studies showed low risk for misclassification of interventions and for deviations from intended interventions. Missing-data bias and outcome-measurement bias were low in every study except Yao 2016, which was rated moderate for D6 (13). Selective-reporting bias was uniformly low. Overall risk of bias was low in 5 studies, moderate in 4, and critical in 1 (23). No significant publication bias was detected by Egger's test (*p* = 0.51) for hospital LOS outcome. The funnel plot did not reveal asymmetry (Supplementary Figure S3).

### 3.6 Meta-regression

There was no significant linear association between the pooled effect on hospital length of stay and the proportion of patients who underwent at least one lobectomy in each cohort; the regression coefficient was non-significant, indicating that lobectomy rate did not explain the between-study variability in this meta-analysis (Supplementary Figure S4).

## 4 Discussion

In this systematic review and meta-analysis of ten observational studies comprising 1,015 patients, the single-stage strategy proved more efficient without compromising safety. Pooled estimates significantly reduced operative time, hospital length of stay, and per-patient costs. Importantly, there was no significant difference between strategies in intraoperative blood loss, persistent air leak, or perioperative arrhythmia, underscoring that the gains in efficiency are not achieved at the expense of increased morbidity. A clinically relevant advantage of the one-stage strategy was the shorter postoperative hospital LOS, with an average reduction of 2.8 days, despite the high heterogeneity observed between studies. Similar magnitudes have been reported in a recent propensity-matched series of synchronous bilateral resections, where median LOS fell from 9 to 5 days when both lungs were addressed in a single sitting (16). A shorter LOS is not merely a logistical convenience; for oncologic patients, it translates into fewer delays before adjuvant chemotherapy or targeted therapy can be started, a benefit that thoracic-oncology guidelines and single-institution experiences have repeatedly highlighted (24–27). Our meta-regression showed that the magnitude of the LOS advantage was independent of surgical complexity (percentage of lobectomies), suggesting that the benefit applies to both limited resections and anatomic lobectomies. Contrary to the intuitive expectation that a simultaneous bilateral resection would be longer, operative time was 24 min shorter in the one-stage cohort. While it might be assumed that performing bilateral resections in a same anesthetic event/same operative session would increase technical complexity and prolong surgery, the pooled data suggest otherwise. A likely explanation is that the two-stage approach inherently duplicates several perioperative steps, including anesthesia induction, safety checks, and patient repositioning, elements that are streamlined in the one-stage setting (28, 29). Previous reports have emphasized that the actual incision-to-closure time may be similar between approaches, but the total time in the operating room is more affected by logistical factors between separate procedures (22, 23, 30). Therefore, the observed reduction in operative duration likely reflects the overall efficiency gained from a single anesthetic event and continuous intraoperative workflow, rather than a purely technical simplification of the procedure. We found no significant differences in prolonged air leak, perioperative arrhythmia, or blood loss. The neutral estimates echo earlier narrative reviews suggesting that perioperative morbidity depends more on pulmonary reserve and extent of parenchymal loss than on staging strategy (31). Importantly, the complication analyses displayed minimal statistical heterogeneity, implying that the absence of excess risk with one-stage surgery is consistent across diverse patient populations and surgical approaches (16, 32). This reassurance is critical when counseling patients who fear an additive physiologic insult from simultaneous bilateral surgery (33). Direct hospital costs were, on average, USD 5,500 lower with a one-stage approach. This mirrors the 25%–35% cost reduction observed in recent propensity score matching cohorts and is largely driven by the avoidance of a second admission, duplicated preoperative work-up, and repeated anesthesia fees (16, 32). In publicly funded systems, such savings may allow re-allocation of resources to novel systemic therapies that improve survival yet strain budgets (34–37). For low- and middle-income countries, the financial argument may be even more compelling than the modest peri-operative advantages (38, 39).

This meta-analysis is constrained by the observational characteristics of the included studies, which carry inherent risks of selection bias and residual confounding. Long-term oncologic outcomes were not consistently documented, hindering a conclusive evaluation of oncologic equivalence. Cost analyses were derived from a limited number of Asian studies, which may restrict the generalizability of economic findings to other healthcare systems. Furthermore, definitions of postoperative complications were inconsistent, and various outcomes exhibited significant statistical heterogeneity, necessitating careful interpretation. Lastly, outcomes were not consistently categorized by the degree of resection, which constrains conclusions in high-risk scenarios, such as bilateral lobectomy conducted during a single anesthetic event.

This meta-analysis is limited primarily by the observational nature of the evidence. Although we performed subgroup and meta-regression analyses, residual confounding, especially the selection of fitter patients for one-stage surgery, cannot be excluded. Second, cost data derived from just three Asian studies; generalizability to other health-care systems is uncertain. Third, outcome definitions (e.g., “prolonged air leak”) were not uniform, which may have diluted true differences. We attempted to mitigate these issues through *a priori* protocol registration, duplicate extraction, analytic transparency, and multiple sensitivity analyses, yet recognize that high-quality randomized trials remain essential to confirm our findings.

## 5 Conclusion

This meta-analysis suggests that, in selected patients, single-stage bilateral thoracoscopic resection is associated with shorter operative time, reduced hospital stay, and lower direct costs compared with a staged approach, without a clear increase in short-term morbidity. However, given the observational design, heterogeneity, and limited stratification by extent of resection, these findings should be interpreted cautiously, and single-stage surgery should be considered on an individualized basis in experienced centers.

## Data Availability

The original contributions presented in the study are included in the article/Supplementary Material, further inquiries can be directed to the corresponding author.
