# Supplementary material for: One-stage vs. two-stage thoracoscopic surgery for synchronous bilateral pulmonary nodules: a systematic review and meta-analysis
Source: Front Surg. 2026 Jan 21;12:1755084. doi: 10.3389/fsurg.2025.1755084 (PMC12868250; doi:10.3389/fsurg.2025.1755084)
Supplement: Supplementary file 1 [file Supplementaryfile1.docx]

**SUPPLEMENTARY MATERIAL**

**TITLE:** One-stage versus two-stage thoracoscopic surgery for synchronous bilateral pulmonary nodules: A systematic review and meta-analysis

**SUPPLEMENTARY TABLES AND FIGURES LEGENDS:**

**Supplementary Table S1.** Search strategy.

**Supplementary Figure S1.** Subgroup analysis of hospital length of stay based on lung lesion etiology.

**Supplementary Figure S2.** Leave-one-out analysis of hospital length of stay.

**Supplementary Figure S3.** Funnel plot of hospital length of stay.

**Supplementary Figure S4.** Meta regression of hospital length of stay using proportion of patients who underwent at least one lobectomy as covariate.

**Supplementary Table S1.** Search strategy.

| PubMed, Embase and Cochrane | (pulmonary nodule OR lung nodule OR pulmonary lesion OR lung lesion OR "Multiple Pulmonary Nodules" OR "Lung Neoplasms" OR "primary lung cancer" OR "primary pulmonary neoplasm" OR "primary lung neoplasms" OR "non-small cell lung cancer" OR NSCLC OR "metastatic pulmonary nodule" OR "pulmonary metastases" OR "lung metastases" OR "secondary lung neoplasms")  **AND** (bilateral OR synchronous OR both lungs)  **AND** (video-assisted thoracoscopic surgery OR VATS OR thoracoscopic OR thoracoscopy)  **AND** (one stage OR single stage OR simultaneous)  **AND** (two stage OR staged) |
| --- | --- |

**Supplementary Figure S1.** Subgroup analysis of hospital length of stay based on lung lesion etiology. CI: confidence interval; IV: inverse variance; SD: standard deviation.

**
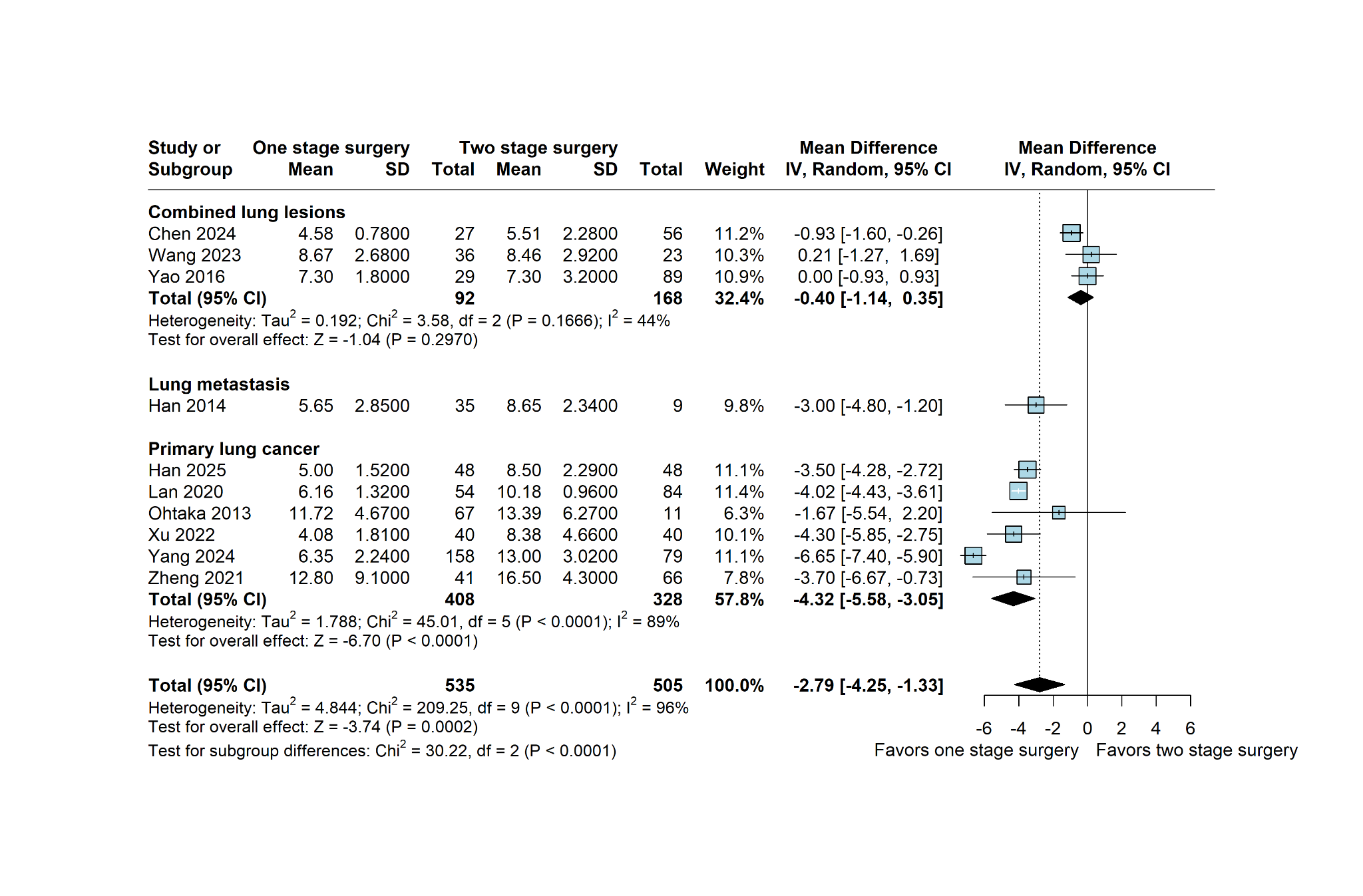
**

**Supplementary Figure S2.** Leave-one-out analysis of hospital length of stay. CI: confidence interval; IV: inverse variance; MD: mean difference.


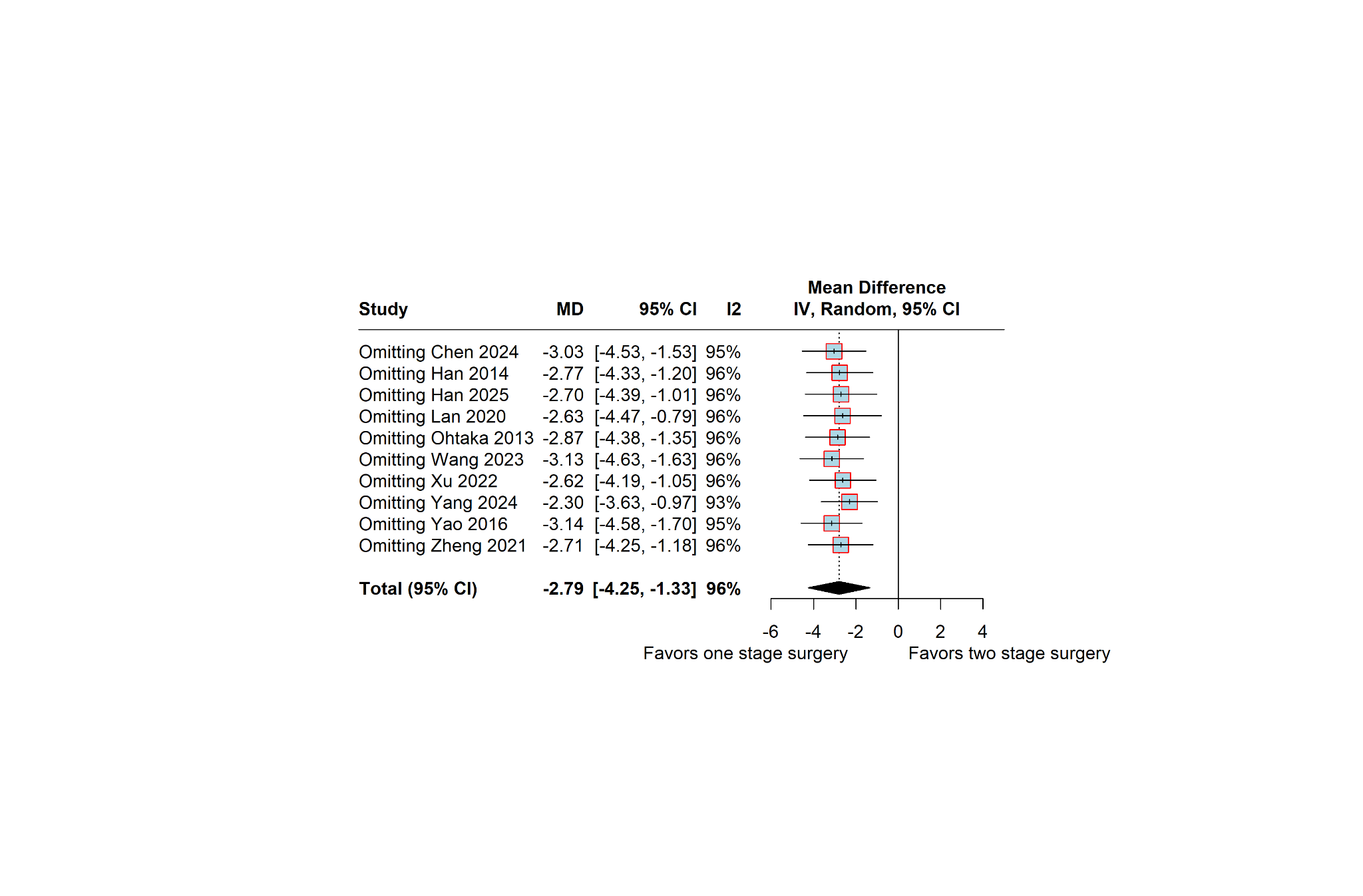


**Supplementary Figure S3.** Funnel plot of hospital length of stay.


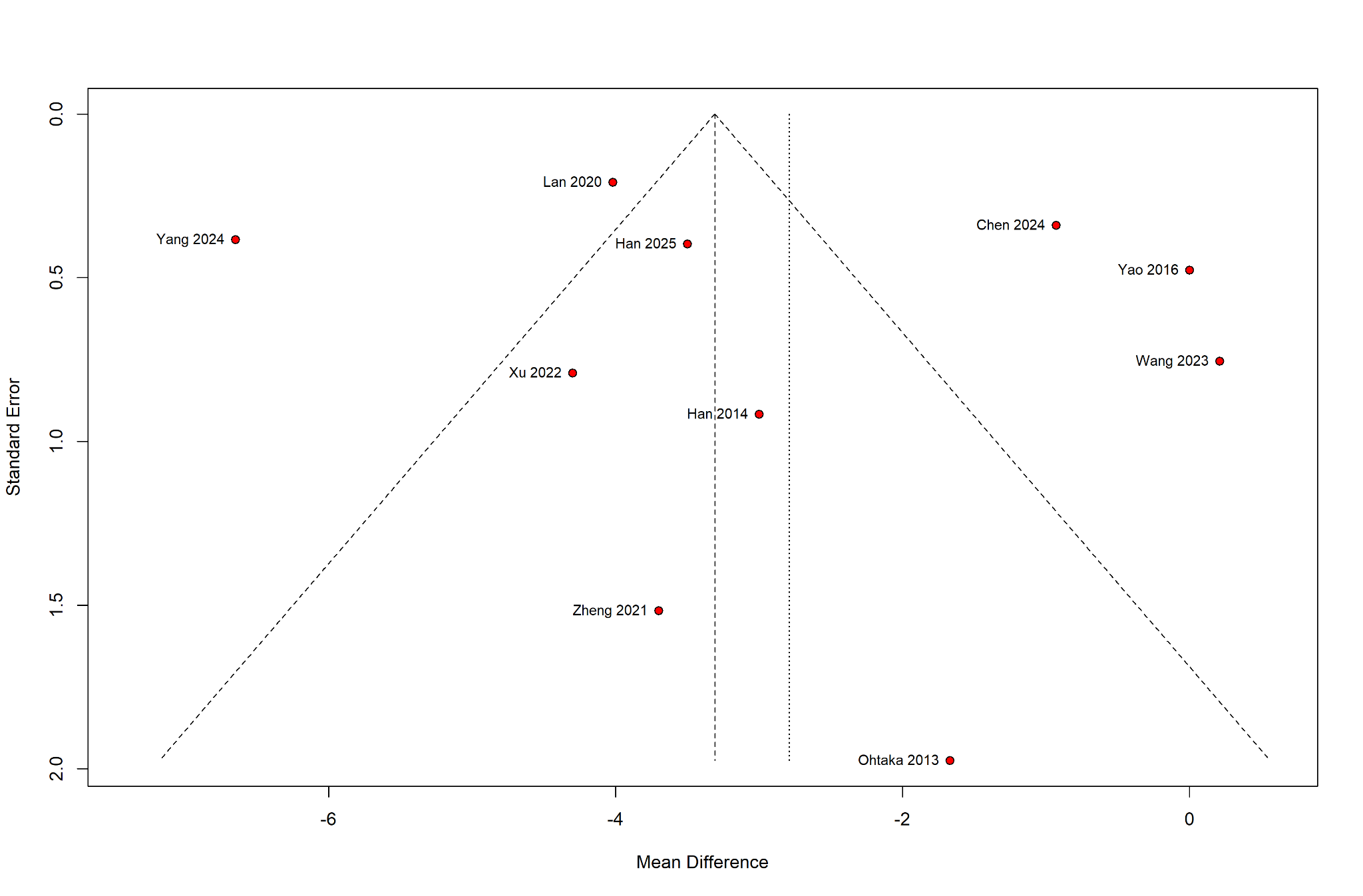


**Supplementary Figure S4.** Meta regression of hospital length of stay using proportion of patients who underwent at least one lobectomy as covariate.


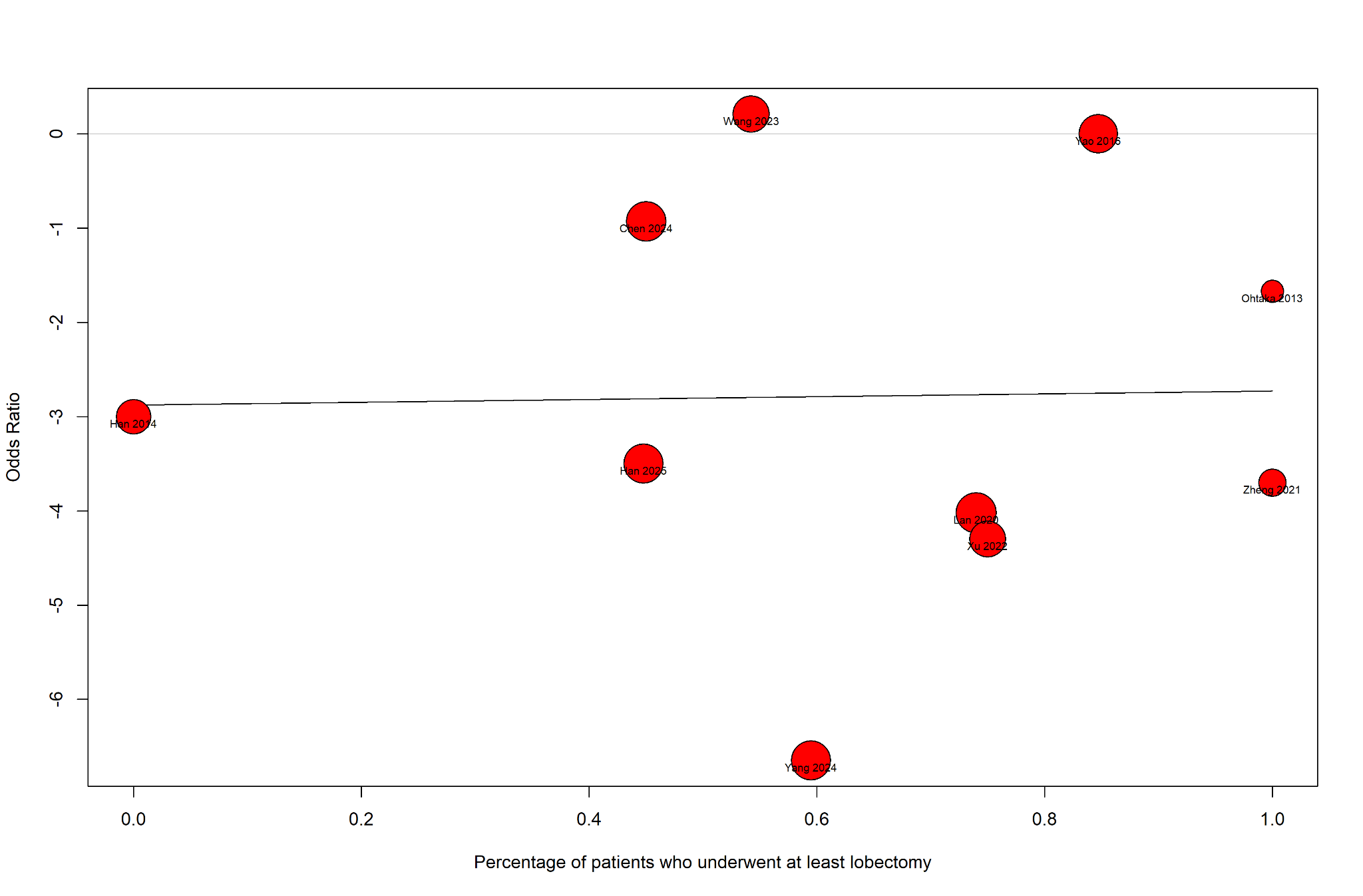


|  | Effect estimate | p-value | I² | Test for residual heterogeneity |
| --- | --- | --- | --- | --- |
| Intercept | -2.8778 | 0.1498 | 96.12% | <0.0001 |
| % of patients who underwent at least lobectomy | 0.1487 | 0.9599 |  |  |
